# Conformation of bis-nitroxide polarizing agents by multi-frequency EPR spectroscopy^†^

**DOI:** 10.1039/c8cp05236k

**Published:** 2018-10-10

**Authors:** Janne Soetbeer, Peter Gast, Joseph J. Walish, Yanchuan Zhao, Christy George, Chen Yang, Timothy M. Swager, Robert G. Griffin, Guinevere Mathies

**Affiliations:** a Francis Bitter Magnet Laboratory and Department of Chemistry, Massachusetts Institute of Technology, Cambridge, MA 02139, USA; b Department of Physics, Huygens-Kamerlingh Onnes Laboratory, Leiden University, PO Box 9504, 2300 RA Leiden, The Netherlands; c Department of Chemistry, Massachusetts Institute of Technology, Cambridge, MA 02139, USA

## Abstract

The chemical structure of polarizing agents critically determines the efficiency of dynamic nuclear polarization (DNP). For cross-effect DNP, biradicals are the polarizing agents of choice and the interaction and relative orientation of the two unpaired electrons should be optimal. Both parameters are affected by the molecular structure of the biradical in the frozen glassy matrix that is typically used for DNP/MAS NMR and likely differs from the structure observed with X-ray crystallography. We have determined the conformations of six bis-nitroxide polarizing agents, including the highly efficient AMUPol, in their DNP matrix with EPR spectroscopy at 9.7 GHz, 140 GHz, and 275 GHz. The multi-frequency approach in combination with an advanced fitting routine allows us to reliably extract the interaction and relative orientation of the nitroxide moieties. We compare the structures of six bis-nitroxides to their DNP performance at 500 MHz/330 GHz.

## Introduction

Dynamic Nuclear Polarization (DNP) is a powerful method to enhance the sensitivity of NMR spectroscopy. In DNP, microwave irradiation induces a polarization transfer from unpaired electrons to nuclei, thereby enhancing the NMR signal. In combination with magic-angle spinning (MAS) NMR, DNP enables structural studies in materials science and biology that would otherwise be out of reach due to insufficient sensitivity. For a recent comprehensive review see Lilly Thankamony *et al*.^1^

In MAS NMR/DNP, the NMR sample is doped with a paramagnetic species, which is often a stable organic radical and called a polarizing agent, and polarization is transferred *in situ* by high-power, high-frequency microwaves. Typically, polarizing agents are dissolved in a glass-forming solvent that is frozen after addition to the molecular system of interest. The frozen glassy matrix assures that dipolar couplings are available for electron–^1^H and ^1^H–^1^H polarization transfer and that the polarizing agents are distributed homogeneously. Performing DNP under cryogenic conditions further enhances the nuclear polarization and enables the study of mobile or transient species by MAS NMR.^2,3^

The DNP mechanism that is currently most successfully used in MAS NMR/DNP applications is the cross-effect.^4–7^ Cross-effect (CE) DNP relies on two weakly interacting electrons, one of which is dipolar coupled to a nearby ^1^H. Hence, for CE DNP, biradicals are the polarizing agents of choice.^8^ In an NMR/DNP-sample spinning at the magic angle, polarization is transferred through energy-level crossings, which occur regularly with each rotor period. Ideally during each rotor period, microwave irradiation first generates a large polarization difference between the two electron spins, which is consecutively transferred to the nuclei during a CE level crossing. During this crossing, the CE matching condition, $\omega_{0,{\text{S}}_{1}}-\omega_{0,{\text{S}}_{2}}\approx \omega_{0{,}^{1}\text{H}}$, which states that the difference between the Larmor frequencies of the two electrons is approximately equal to the ^1^H Larmor frequency, is momentarily fulfilled.^9,10^

Both the chemical structure of the polarizing agent and its local environment, which is typically determined by the solvent matrix, play a critical role in the DNP enhancement. For CE DNP, a multitude of bis-nitroxide radicals have been synthesized and their DNP performance has been investigated.^11–22^ Clearly much progress has been made and ^1^H NMR signal enhancement factors > 200 have become the standard at 400 MHz/263 GHz.

Remarkably, it is not well understood why certain bis-nitroxides are more efficient polarizing agents than others or why certain solvent matrices lead to higher enhancements than others. Several factors likely play a role. First, the degenerate state mixing responsible for the polarization transfer during a CE level crossing is proportional to $-(D+2J)A_{xz{,}^{1}\text{H}}/\omega_{0{,}^{1}\text{H}}$, where *D* is the electron-electron dipolar interaction, *J* is the Heisenberg exchange interaction, which is roughly determined by the wave function overlap of the two unpaired electrons, and $A_{xz{,}^{1}\text{H}}$ is the strength of the electron-^1^H dipolar interaction.^9,23–25^ Thus, within certain boundary conditions, an increased strength of any of these interactions is expected to lead to an improved DNP efficiency. Second, there is likely to be a preferred relative orientation for the two nitroxide moieties, such that the CE matching condition can be fulfilled at the right moment during the rotor period.^24,26–29^ A rigid chemical linker, which locks the two nitroxides into their optimal position and orientation, may therefore be advantageous.^12,15^ Third, the bis-nitroxide needs to dissolve well in the solvent matrix, the choice of which may depend on the application in biology or material science. The solvent matrix may also determine the exact conformation of the bis-nitroxide and the availability of ^1^H for transfer of polarization to the solvent and the molecular system of interest. Fourth, electronic relaxation rates determine the electron polarization difference that can be established and thereby the CE DNP enhancement.^16,20,30–32^ Relaxation rates may depend on the chemical structure of the polarizing agent and the selected solvent matrix.^33,34^

**Scheme 1:**
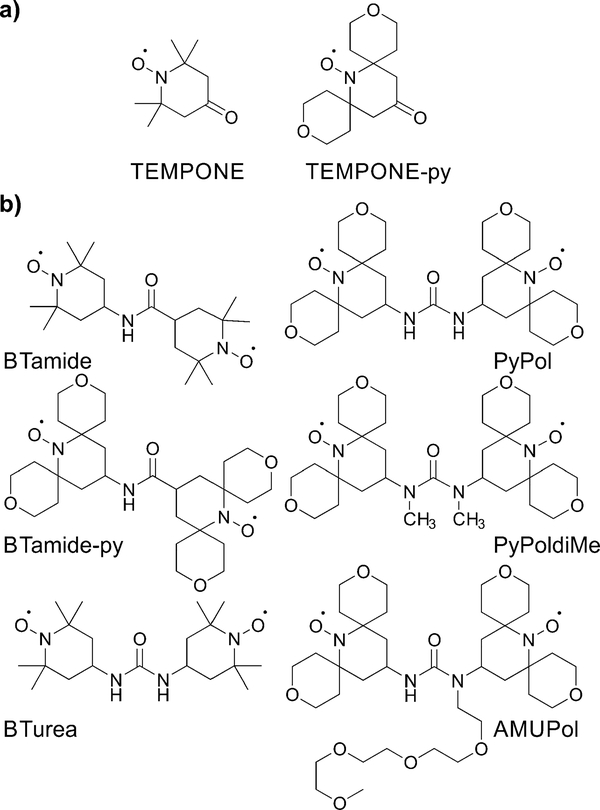
Chemical structures of (a) the mono-nitroxides and (b) the bis-nitroxide polarizing agents investigated in this study.

Here we determine the conformations of six bis-nitroxide polarizing agents (Scheme 1) in a frozen glassy matrix relevant for MAS NMR/DNP using multi-frequency EPR spectroscopy. This method allows us to extract the electron-electron dipolar interaction, the Heisenberg exchange interaction, as well as the relative orientation and position of the two nitroxide moieties. For each bis-nitroxide we have chosen a solvent matrix in which it shows fair to good solubility, *i.e.* BTamide, BTamide-py, and BTurea^26^ are investigated in a mixture of DMSO and water, PyPol,^17^ PyPoldiMe,^19,20^ and AMUPol^17^ in a mixture of glycerol and water. We compare the properties of the six bis-nitroxides to their DNP performance in the corresponding matrices at 500 MHz/330 GHz.

## Materials and methods

### Synthesis of the polarizing agents

TEMPONE was purchased from Sigma Aldrich (St. Louis, MO), and TEMPONE-py was prepared following a procedure reported by Sakai *et al.*^35^ The bis-nitroxide BTurea was synthesized as reported in ref. 26. AMUPol, PyPoldiMe, and PyPol were synthesized as described in ref. 17 and 20.

**Scheme 2:**
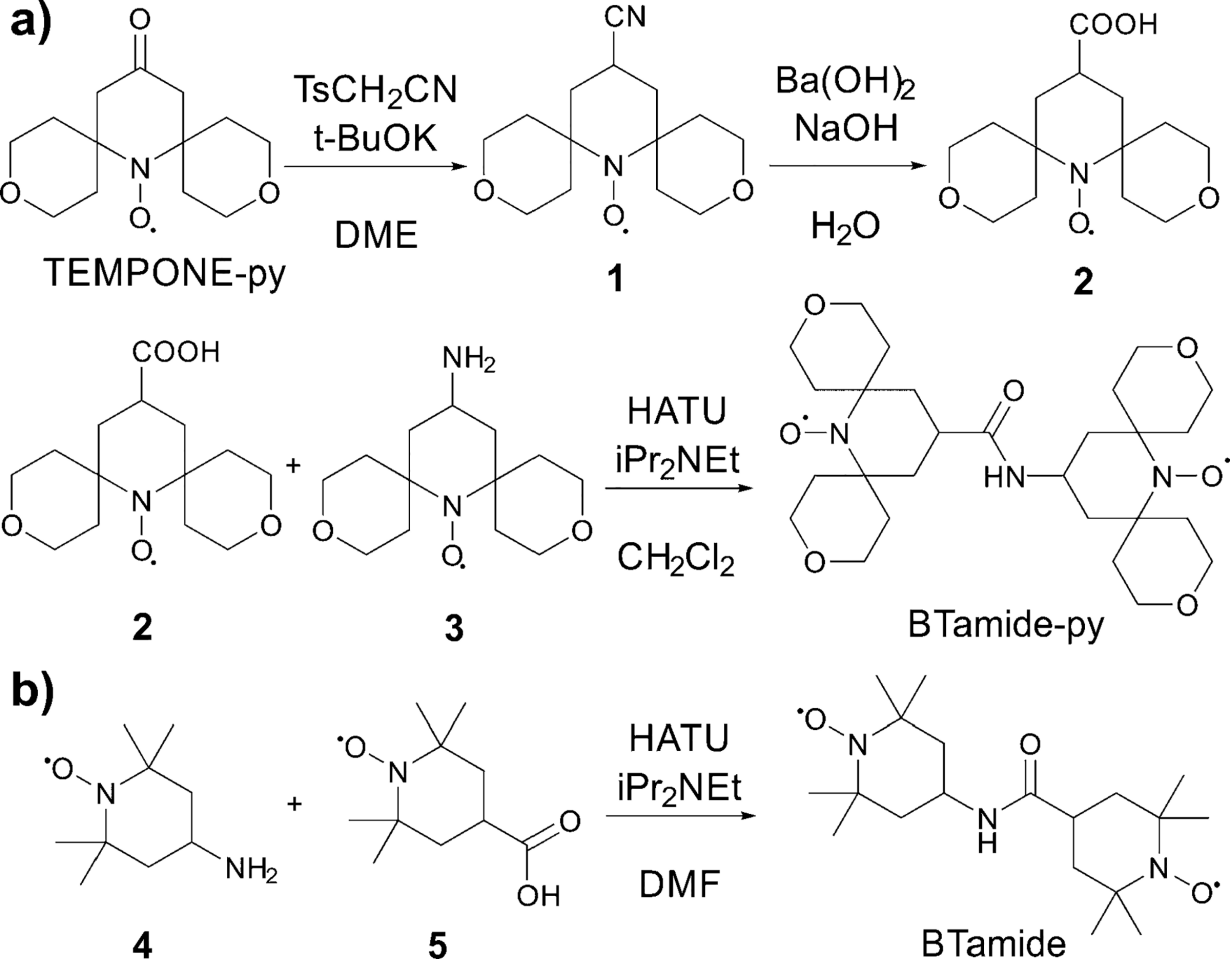
Chemical synthesis of (a) BTamide-py from TEMPONE-py and (b) BTamide from TEMPONE.

The synthesis of BTamide-py is shown in Scheme 2a. First, nitroxide **2** was prepared following a procedure reported by Rauckman *et al*.^36^ To a solution of TEMPONE-py (300 mg, 1.18 mmol) and tosyl methyl isocyanide (322 mg, 1.65 mmol, 1.40 equiv.) in DME (12 mL) at 0 °C, was added a solution of potassium *tert*-butoxide (397 mg, 3.54 mmol, 3.0 equiv.) in DME (3 mL) and *tert*-butanol (3 mL). After stirring at 0 °C for 1 h, the mixture was allowed to warm to room temperature and stirred overnight. Water was then added and the mixture was extracted with diethyl ether (20 mL × 3). The combined organic layers were dried over anhydrous sodium sulfate. After the solvent was removed under vacuum, nitroxide **1** (200 mg, 0.75 mmol, yield: 64%) was obtained as a red solid and used without further purification. To a solution of nitroxide **1** (200 mg, 0.75 mmol) in methanol was added a solution of barium hydroxide and sodium hydroxide in water. The mixture was refluxed for 2 hours before it was cooled to room temperature and extracted with chloroform. The aqueous solution was acidified with HCl (0.5 N) and extracted with chloroform. The solvent was removed under vacuum and the residue was purified by silica gel chromatography (the solvent was CH_2_Cl_2_ with 1% MeOH) to give nitroxide **2** (148 mg, 0.53 mmol, yield: 69%) as a red solid. HRMS (ESI): *m/z* calculated for C_14_H_22_NO_5_ [M + H]^+^: 285.1571; found: 285.1570. For the last step, nitroxide 3 was prepared following a procedure reported by Sauvée *et al.*^17^ Then, *N*,*N*-diisopropylethylamine (9 mg, 0.07 mmol, 2.0 equiv.) was added to a solution of nitroxide **2** (10 mg, 0.035 mmol), nitroxide **3** (11 mg, 0.042 mmol, 1.2 equiv.), and 1-[bis(dimethylamino)methylene]-^1^*H*-1,2,3-triazolo[4,5-*b*]-pyridinium 3-oxid hexafluorophosphate (HATU, 27 mg, 0.071 mmol, 2.0 equiv.) in CH_2_Cl_2_ (2 mL). The mixture was stirred at room temperature overnight (16 h). The solvent was removed under vacuum and the residue was purified by silica gel chromatography (the solvent was CH_2_Cl_2_ with 1% MeOH) to give BTamide-py (12 mg, 0.023 mmol, yield: 66%) as a red solid. HRMS (ESI): *m/z* calculated for C_27_H_44_N_3_O_7_ [M + H]^+^: 522.3174; found: 522.3165.

The synthesis of BTamide is shown in Scheme 2b. At room temperature, 4-carboxy-TEMPO (nitroxide 5, 40 mg, 0.2 mmol), *N*,*N*-diisopropylethylamine (0.216 mL, 1.24 mmol, 6.2 equiv.), and 4-amino-TEMPO (nitroxide **4**, 0.058 g, 0.34 mmol, 1.7 equiv.) were added to a pre-dried Schlenk-flask containing 5.4 mL of dry dimethylformamide under argon atmosphere. The resulting solution was stirred and cooled in an ice bath to 0 °C. HATU (91 mg, 0.24 mmol, 1.2 equiv.) was added under argon atmosphere and the solution was stirred for 5 minutes at 0 °C. The solution was allowed to return to room temperature and after one hour the solvent was removed under vacuum. The resulting material was then purified *via* silica-gel column chromatography (the solvent was a 1 : 1 mixture of ethyl acetate and hexane) to give 64 mg of the BTamide. HRMS (ESI): *m/z* calculated for C_19_H_35_N_3_O_3_(2e^−^) [M^−^ + 2H]^+^: 355.2829; found: 355.2826.

### Spectroscopic sample preparation

For the DNP experiments, a bis-nitroxide was dissolved in a heavily deuterated glass-forming matrix to form a 10 mM solution. This was either d_8_-glycerol: D_2_O: H_2_O 60:30:10 v:v:v for PyPol, PyPoldiMe, and AMUPol or d_6_-DMSO: D_2_O: H_2_O 60:30:10 v:v:v for BTamide, BTamide-py, and BTurea. Uniformly ^13^C-labeled urea was added to a 1 M concentration.

For the EPR experiments at cryogenic temperatures, 1 mM solutions of a bis-nitroxide were prepared through dissolution in fully deuterated DMSO/water or glycerol/water. Deuterated glycerol was obtained from DyNuPol (Cambridge, MA), deuterated DMSO was obtained from Cambridge Isotope Laboratories, Inc. (Cambridge, MA).

### EPR spectroscopy

#### 9.7 GHz (X band)

Continuous-wave (CW) X-band frozen solution EPR spectra were obtained on a Bruker ElexSys E580 spectrometer using the ER4118X-MD4 probe with a dielectric resonator and sample tubes of 4 mm OD. A temperature of 80 K was maintained using a CF 935 flow cryostat with liquid nitrogen as a cryogen and an ITC 503S temperature controller (Oxford Instruments). The microwave power was 0.001 mW (53 dB attenuation), the modulation amplitude was 0.05 mT, the modulation frequency was 100 kHz, and the microwave frequency was 9.7056 GHz.

#### 140 GHz (D band)

D-band EPR spectra were obtained on a spectrometer constructed by Smith *et al.* operating at a fixed microwave frequency of 139.997 GHz.^37^ Coherent pulses as well as continuous-wave (CW) microwaves are available at a power level of ~100 mW generated by a Virginia Diodes (Charlottesville, VA) active multiple chain (AMC). A silver TE_011_ resonator focusses the microwave field over a sample volume of approximately 200 nL. Echo-detected (ED) EPR spectra were recorded with a Hahn echo sequence: (π/2)_X_ – τ − (π)_X_ − τ − echo with 90° pulses of 40 ns and τ = 500 ns using a two-step phase cycle. At each field position 400 shots were acquired with a repetition time of 1 ms. Samples were kept at 80 K using liquid nitrogen, an Oxford Spectrostat CF flow cryostat, and an ITC 502 temperature controller (Oxford Instruments). A Resonance Research (Billerica, MA) field-mapping unit (FMU) measures the ^1^H resonance frequency of a water sample placed just below the cryostat in the magnet bore.^38^

#### 275 GHz (J band)

J-band EPR spectra were obtained on a spectrometer constructed by Blok *et al*.^39–41^ The operating frequency is 275.7 GHz and the maximum microwave power output is approximately 1 mW. A single-mode TE_011_ resonator focusses the microwave field over a sample volume of approximately 20 nL. CW spectra were obtained at approximately 1 μW of microwave power, with a modulation amplitude of 0.15–0.35 mT, and a modulation frequency of 1.8 kHz. Samples were kept at 80 K using liquid helium as a cryogen, an Oxford Spectrostat CF flow cryostat, and an ITC 503S temperature controller (Oxford Instruments).

#### 500 MHz/330 GHz MAS NMR/DNP

DNP/MAS NMR experiments were performed on a custom-designed DNP-NMR spectrometer operating at 11.73 T/328.956 GHz/499.450 MHz. Cryogenic DNP/MAS NMR experiments were performed using a homebuilt 3.2 mm triple-resonance (^1^H, ^13^C, and ^15^N) probe as described by Reese *et al*.^42^ Microwaves were generated by a homebuilt gyrotron operating at the second harmonic of the electron cyclotron frequency.^43^ Oversized, helically corrugated waveguides were employed to transfer microwaves with minimum losses. Optimal output power from the gyrotron was 12.6 W and the microwave power at the NMR sample is estimated to be 9.1 W.

The DNP signal enhancement (ε_on/off_) due to polarization transfer from e- to ^1^H was observed indirectly on ^13^C using cross-polarization (CP). CP was established with a nutation frequency of 50 kHz on ^1^H, and 45 kHz on ^13^C. A spinning frequency of 5.2 kHz was used. Two-pulse phase modulation (TPPM) decoupling at an amplitude of 83 kHz was applied during acquisition. The recycle delay was 4 s. Microwave radiation was continuously applied throughout the experiment. Sapphire rotors were used because of their superior microwave transmission in comparison to their zirconia counterparts. The temperature at the rotor during acquisition was controlled at approximately 85 K using a homebuilt heat exchanger.

### Multi-frequency EPR analysis

The spin Hamiltonian that describes a nitroxide biradical is given by

(1) $$H=\mu_{\text{B}}B_{0}g_{1}S_{1}-\gamma_{\text{n}}B_{0}I_{1}+S_{1}A_{1}I_{1}+\mu_{\text{B}}B_{0}g_{2}S_{2}-\gamma_{\text{n}}B_{0}I_{2}+S_{2}A_{2}I_{2}\;\;+S_{1}DS_{2}-2JS_{1}S_{2}$$

**Fig. 1:**
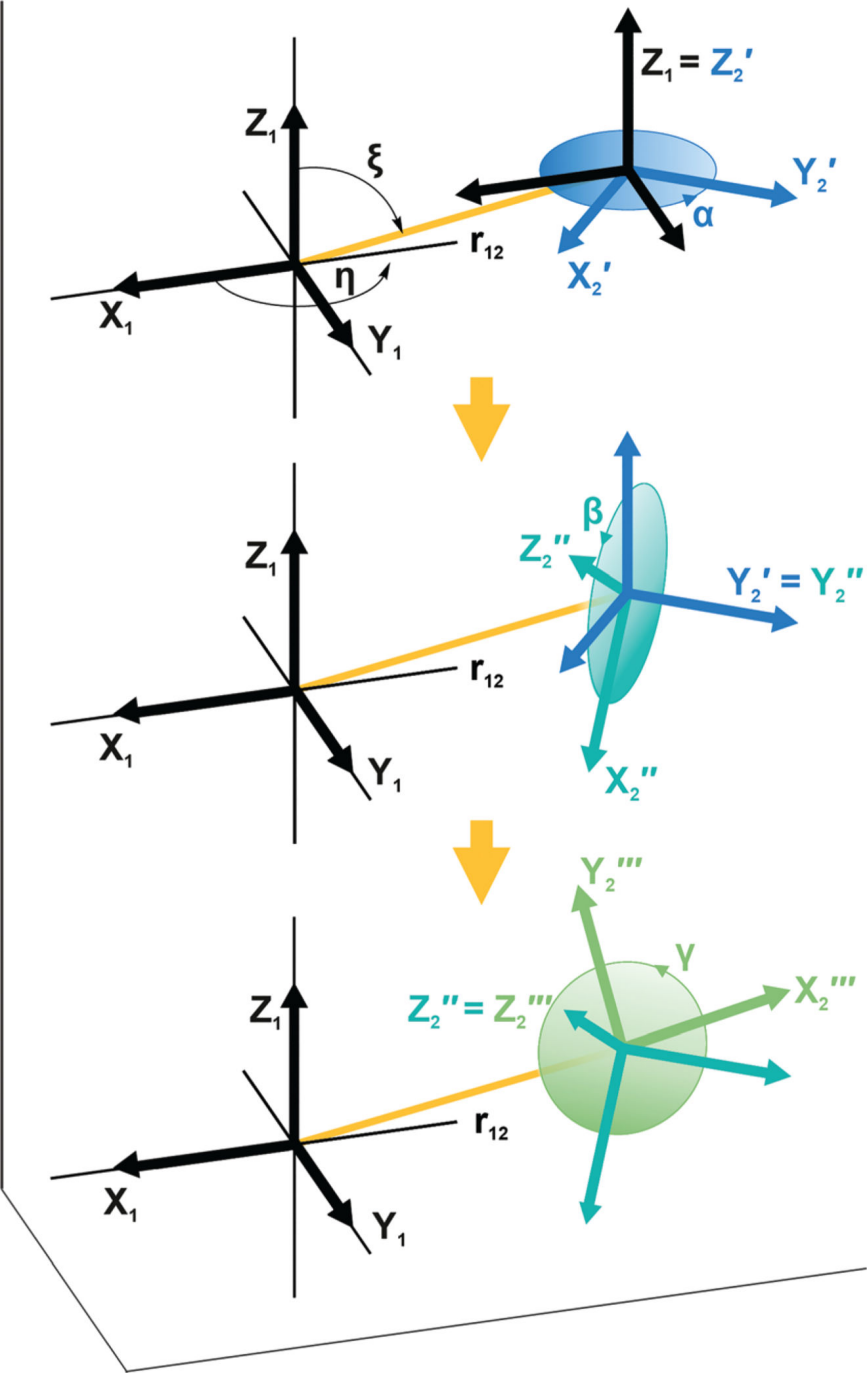
Illustration showing the relative orientation (*α*, *β*, *γ*) and position (*η*, *ξ*, 0) of the *g*- and hyperfine tensors of nitroxide moieties **1** and **2** and the interelectron vector *r*_12_ for AMUPol (see Table 2). Within each TEMPONE moiety the *g*- and hyperfine principal axes are directed as follows: *x* is along the N-O bond, *y* is in the C(NO)C-plane perpendicular to *x*, and *z* points out of the C(NO)C-plane.^42,43^ Note that the exact alignment of the principal axes may vary with the conformation and is affected by solvent and substituents.^45–47^

The first six terms are the electron Zeeman term with *μ*_B_ the Bohr magneton and ***B***_0_ the magnetic field, the nitroxide ^14^N nuclear Zeeman term, and the ^14^N-electron hyperfine interaction, for nitroxides **1** and **2**. For TEMPO-like nitroxide radicals, the principal axes of the *g-* and hyperfine tensors approximately coincide.^44,45^ The relative orientation of the tensors of nitroxides **1** and **2** is determined by the chemical structure of the biradical and described by the Euler angles *α*, *β*, *γ*, see Fig. 1. The tensor ***g***_2_ in the principal axes system of the tensor ***g***_1_ is expressed in its own principal axes system by

(2) $$g_{2}^{\text{PAS},2}=R_{12}(\alpha ,\beta ,\gamma )g_{2}^{\text{PAS},1}{R_{12}}^{-1}(\alpha ,\beta ,\gamma )$$

The transformation is analogous for the hyperfine tensor.

(3) $$A_{2}^{\text{PAS},2}=R_{12}(\alpha ,\beta ,\gamma )A_{2}^{\text{PAS},1}{R_{12}}^{-1}(\alpha ,\beta ,\gamma )$$

The rotation matrix ***R***(*α*, *β*, *γ*) describes three consecutive counter-clockwise rotations of the frame around the axes *Z*, *Y′,* and *Z′′*, as shown in Fig. 1, following the convention of Rose.^46^

The last two terms in eqn (1) describe the electron–electron interaction. ***D*** is a traceless tensor that contains the anisotropic interactions, while the parameter −2*J* is due to the isotropic Heisenberg exchange interaction. Assuming that ***D*** arises solely from the electron-electron dipolar interaction and that the point-dipole approximation is valid,^47^ only the secular terms are relevant^48^ and we can write ***D***, in its own principal axes system, as

(4) $$D^{\text{PAS},\text{D}}=\frac{\mu_{0}}{4\pi \hbar }\frac{g_{1}g_{2}\mu_{\text{B}}^{\;2}}{r_{12}^{\;3}}\left(\begin{array}{ccc} -1 & 0 & 0\\ 0 & -1 & 0\\ 0 & 0 & 2 \end{array}\right)$$

Here *r*_12_ is the electron-electron distance and *g*_1_ = *g*_2_ = 1/3(*g*_*x*_ + *g*_*y*_ + *g*_*z*_) the isotropic *g*-value for TEMPONE(-py). The ***D*** tensor in the principal axes system of nitroxide **1** is expressed in its own principal axes system by

(5) $$D^{\text{PAS},\text{D}}=R_{1\text{D}}(\eta ,\xi ,0)D^{\text{PAS},1}{R_{1\text{D}}}^{-1}(\eta ,\xi ,0)$$

The angles *η* and *ξ* give the relative position of the tensors of nitroxides **1** and **2** as illustrated in Fig. 1.

For electron-electron distances ≲25 Å, the distance and relative orientation of the *g*- and hyperfine tensors of the two unpaired electrons can be determined by analyzing the continuous-wave (CW) frozen-solution EPR spectra of the bis-nitroxides at multiple microwave frequencies.^26,49^ Here we perform a global fit of experimental EPR spectra of six bis-nitroxides recorded at X band, D band, and J band. Spectra are calculated by full diagonalization of the spin Hamiltonian using the EPR simulation package EasySpin.^50,51^ Parameters *α*, *β*, *γ*, *η*, *ξ*, *r*_12_, *J*, and *g*_*x*_ are varied to minimize the mean square deviation *χ*^2^ given by

(6) $$\chi_{i}^{2}\left(\alpha ,\beta ,\gamma ,\eta ,\xi ,r_{12},J,g_{x}\right)\;=\frac{1}{N}\sum_{i=1}^{N}{\left[S_{j}^{\mathrm{sim}}\left(\alpha ,\beta ,\gamma ,\eta ,\xi ,r_{12},J,g_{x}\right)-S_{j}^{\exp}\right]}^{2}$$

with the simulated spectrum *S*^sim^, the experimental spectrum S^exp^, and the number of data points *N* in a spectrum at *i* = X, D or J band. The term 1/*N* assures equal weighting of the spectra at the three microwave frequencies in the fitting procedure. To assess the quality of the global fit for each bis-nitroxide we define *χ*_tot_^2^ = *χ*_x_^2^ + *χ*_d_^2^ + *χ*_j_^2^.

To sample the large eight-dimensional parameter space efficiently, the simulated annealing technique was used.^52^ The algorithm, which was implemented in Matlab (The MathWorks, Inc., Natick, MA), generates new parameter test sets by varying the eight fitting parameters sequentially and randomly. The extent of the search for a new parameter set is proportional to the emulated annealing temperature. New parameter sets are always accepted if they lead to a smaller *χ*^2^. However, parameter sets that raise *χ*^2^ may also be accepted according to a Boltzmann-type probability distribution determined by the annealing temperature. Hereby the algorithm avoids becoming trapped in local minima. As the temperature is gradually lowered over the course of the calculation, the extent of the parameter search is dynamically adjusted to maintain an acceptance probability of about 50%.

The full minimization procedure was nevertheless very time consuming. To reduce calculation times, we employed the following measures. (1) Spectra at the three frequencies were calculated in parallel on the Euler High Performance Computing cluster at ETH Zürich. (2) Each proposed set of Euler angles was tested prior to spectrum calculation to see if it corresponds to a chemically reasonable structure, *i.e*. no occurrence of steric clashes. (3) For each polarizing agent a database was generated on-the-fly to avoid computationally expensive recalculation of previously considered parameter combinations. In the optimized simulation procedure, 2000 parameter sets were evaluated in one run over the course of ~1.5 days. After each run, the simulated annealing procedure was reinitiated with the best available parameter set as assessed by the spectroscopist. The number of re-initiations was typically ≥10.

After the simulated annealing procedure, the match between simulated and experimental spectra was further improved by adjusting the isotropic line broadening as well as a the *g*-strain factor of *f* × (|*g*_*x*_ − *g*_*e*_|,|*g*_*y*_ − *g*_*e*_|,|*g*_*z*_ − *g*_*e*_|). The local environment of the minima was investigated by plotting one-dimensional cuts through the high-dimensional *χ*^2^-function for each of the eight optimized parameters. With help of these 1D *χ*^2^-plots, the parameters were further optimized manually, not only aiming to minimize *χ*_tot_^2^, but also to match spectral features in simulation and experiment and ensuring that *χ*_X_^2^, *χ*_D_^2^, and *χ*_j_^2^ displayed common minima. Because the parameters *r*_12_ and *J* are not independent (a shorter distance is likely accompanied by a stronger exchange interaction), a 2D *χ*^2^-plot was used to evaluate the minima for these parameters. To estimate the uncertainty in each parameter for each polarizing agent individually, the minima in the 1D *χ*^2^-plots were fitted with a Gaussian expression up to 2.25*χ*_min_^2^. Subsequently, the standard deviation for *α*, *β*, *γ*, *η*, *ξ*, *g*_*x*_ was computed from the fitted full-width-half-maximum (FWHM) value. For *r*_12_ and *J*, the estimated uncertainty was provided by the width of an ellipse fitted to the 1.25*χ*_min_^2^ level of the 2D *χ*^2^-plot.

Before the experimental spectra were inserted into the fitting algorithm, we followed a number of preparatory steps. First, a baseline correction was applied to all spectra. Second, the D-band spectra, which were recorded using an echo-detected field sweep, are converted into field-modulated (CW) spectra using the function fieldmod in EasySpin based on the procedure by Hyde *et al.* with a virtual modulation amplitude of 0.5 mT.^53^ Third, on the J-band spectrometer, the strength of the external magnetic field is known to ±2 mT. A higher precision in the field position is needed for accurate global fitting of the EPR spectra at all three microwave frequencies. We therefore aligned the J-band spectra with the D-band spectra (for which the field position is accurately known) by the position of *g*_*z*_. This procedure is illustrated in Fig. SI-1 (ESI†). Fourth, one of the two nitroxide moieties in BTamide and BTamide-py is somewhat unstable against reduction over time. Fractions of mono-nitroxides were discernable in their X-, D-, and J-band spectra. These mono-nitroxide contributions were removed following a procedure illustrated in Fig. SI-2 (ESI†).

## Results

**Fig. 2:**
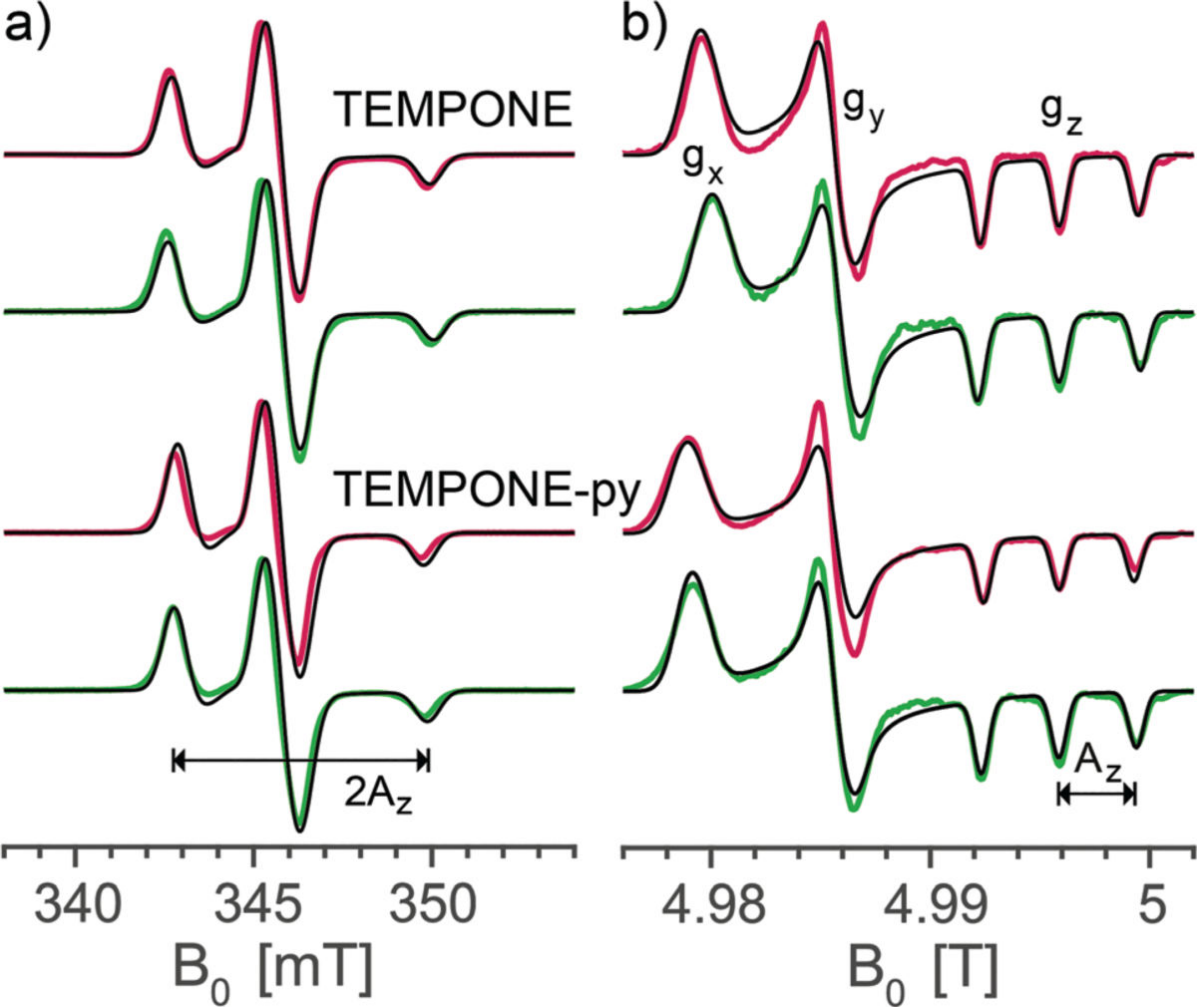
Experimental frozen solution EPR spectra of mono-nitroxides TEMPONE and TEMPONE-py in DMSO/water (red) and in glycerol/water (green) along with simulations (black) at (a) X band (9.7056 GHz) and (b) D band (139.997 GHz).

The principal values of the *g* and hyperfine tensors of a nitroxide radical depend on its molecular structure and environment. For example, for proxyl radicals, the values of *A*_*z*_ and *g*_*x*_ vary depending on whether the nitroxide carries zero, one, or two hydrogen bonds.^54–57^ To obtain starting values for the simulations of the EPR spectra of the bis-nitroxides (see Scheme 1b) of *A*_*z*_, *g*_*x*_, as well as of isotropic line broadening and *g*-strain, we obtained EPR spectra of mono-nitroxides TEMPONE (four β-methyl groups) and TEMPONE-py (two tetrahydropyran rings, see Scheme 1a) both in glycerol/water and DMSO/water. The resulting spectra are, together with simulations, shown in Fig. 2.

The values of *g*_*x*_ indeed depend on solvent matrix and substituents, as is clear from shifts of the *g*_*x*_-peak in the D-band spectra. The observed values of *A*_*z*_ vary concomitantly, but in opposite direction, *i.e.* a decreased value of *g*_*x*_ is accompanied by an increased value of *A*_*z*_, an effect documented in the literature.^58,59^ Simulations were optimized using the fitting routine available in EasySpin and parameters are reported in Table 1.

**Fig. 3:**
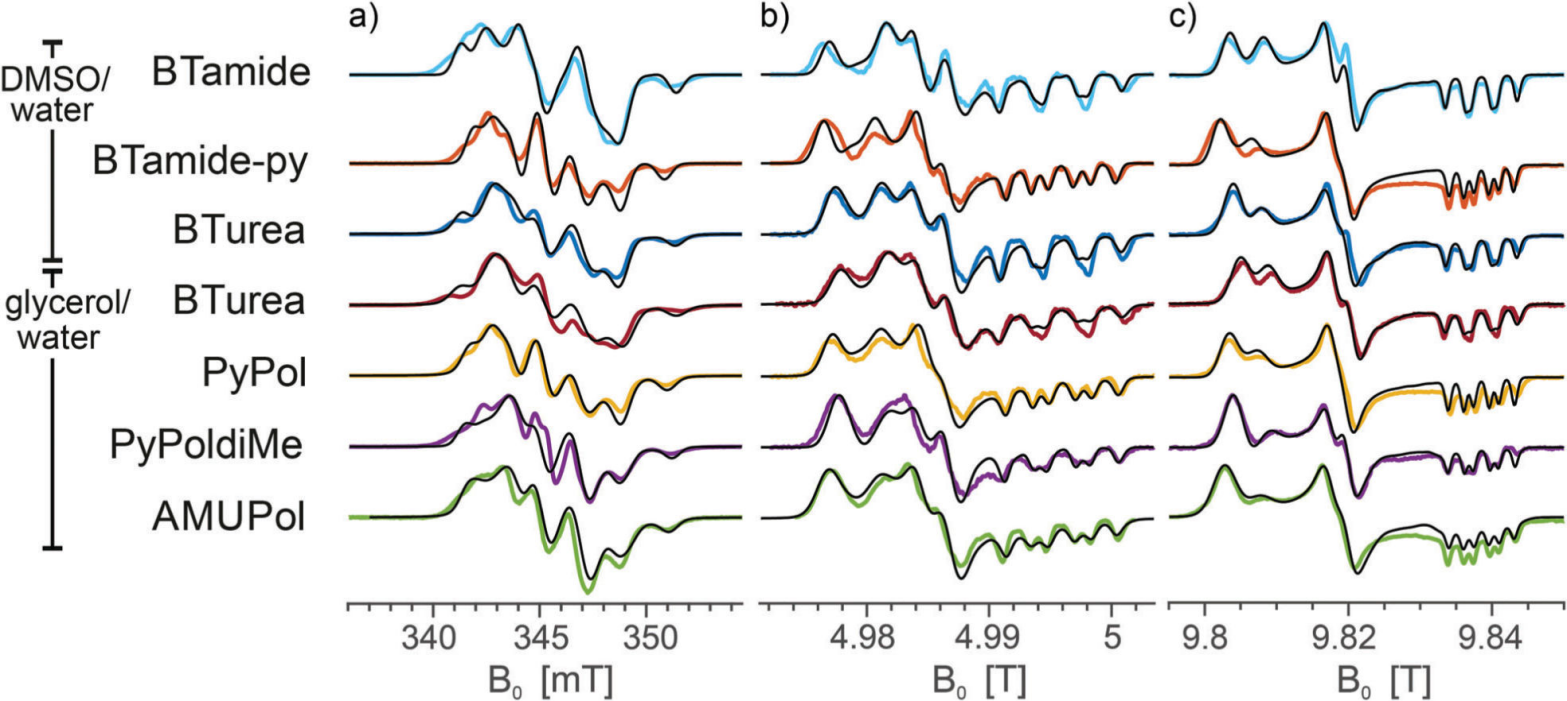
Experimental EPR spectra of frozen solutions of BTamide, BTamide-py, and BTurea in DMSO/water and BTurea, Pypol, PypoldiMe, and AMUPol in glycerol/water (color) along with simulations (black) at (a) X band (9.706 GHz), (b) D band (139.997 GHz), and (c) J band (275.7 GHz). Simulation parameters are specified in Tables 1 and 2.

Fig. 3 shows the frozen solution EPR spectra (colored curves) of six bis-nitroxides (Scheme 1b) at X, D, and J band. In spite of the similar chemical structures, the spectra are clearly distinct. Moreover, small differences between the spectra of BTurea in glycerol/water and in DMSO/water point out that the solvent matrix affects the conformation. Compared to the spectra of the mono-nitroxides, additional splittings and features arise due to electron-electron dipolar coupling and Heisenberg exchange interaction, see Fig. SI-1 (ESI†). In the mono-nitroxide spectra at D band (Fig. 2b), the *g*_*z*_ line is split into three equidistant lines, each separated by the *z*-principle value of the ^14^N hyperfine interaction, *A*_*z*_. Each of these three lines splits further under the influence of electron-electron interaction into six lines, or four in the case of BTamide, where the effective electron-electron splitting is approximately equal to *A*_*z*_ (Fig. 3b and c). Along the *x*- and *y*-principal axes, the ^14^N hyperfine splitting is not resolved in the spectra, but doublets due to electron-electron interactions are evident in the bis-nitroxide D- and J-band spectra. For example, in the D-band spectrum of BTurea in glycerol/water, this doublet is made up of the peaks at 4.977 T and 4.981 T along *x* and of the peaks at 4.984 T and 4.986 T along *y* (analogous in the J-band spectrum: 9.804 T and 9.808 T along *x* and 9.817 T and 9.820 T along *y*).

The observed splittings and detailed lineshapes of the spectra at all three microwave frequencies are determined by the exact molecular conformation of the bis-nitroxide. Specifically, they are sensitive to the distance between the unpaired electrons, *r*_12_, the exchange interaction, *J*, and the relative orientation and position of the two nitroxides described by the five independent angles *α*, *β*, *γ*, *η*, *ξ*. A quantitative interpretation requires a global lineshape analysis by means of an advanced fitting routine, as described in the Materials and methods section. The simulations in Fig. 3 (black curves) are the outcome of this procedure.

The parameters of the simulations in Fig. 3 are summarized in Table 2. Nitroxide-nitroxide distances do not vary much and are between 11.0–11.3 Å (corresponding to dipolar couplings of 39.1–36.1 MHz), except for the outlier BTamide, which has a considerably shorter distance of 10.4 Å (46.3 MHz). The values of the exchange interaction show more variation, with values between −10.1 MHz for BTamide-py and −19.2 MHz for PyPoldiMe. The relative orientations and positions vary for the six bis-nitroxides we investigated, but nevertheless display a common pattern. *η* is around 180°, while *ξ* is around 90°. *α* + *γ* is around 180°. *β* varies in a broad range around 90° with AMUPol having the smallest value (54°) and PyPoldiMe the largest (125°).

The *g*_*x*_ principal value entered the fitting routine as a variable. For all bis-nitroxides, *g*_*x*_ was found to be larger than *g*_*x*_ of the corresponding mono-nitroxide, with particularly AMUPol showing a large increase. Linewidths were optimized manually in an iterative process with the fitting procedure. Addition of *g*-strain improved the quality of the simulations at D- and J-band. Compared to TEMPONE-py in glycerol/water, the *g*-strain factor has increased for PyPol, PyPoldiMe, and most severely for AMUPol, which has a *g*-strain factor, *f,* of 0.13. For BTurea, PyPol, and AMUPol, an additional Lorentzian isotropic line broadening improved the match between the simulations and the experimental spectra at X band, particularly in the low-field region.

**Fig. 4:**
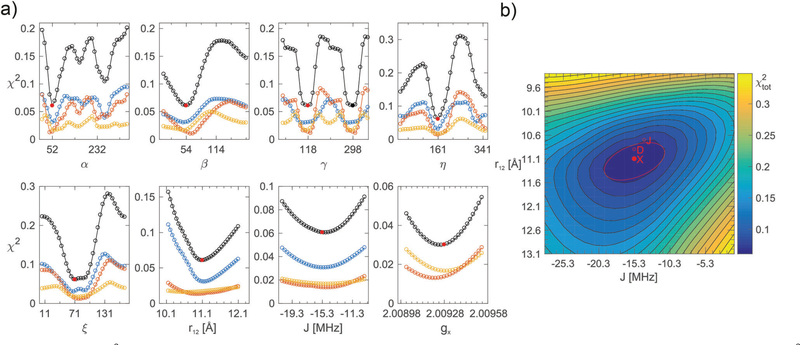
(a) Fitting errors *χ*^2^ as a function of *α*, *β*, *γ*, *η*, *ξ*, *r*_12_, *J*, and *g*_*x*_ for AMUPol in DNPjuice. The solid, red circles indicate the global minimum of *χ*_tot_^2^ (black curve) at a value of 0.061 (see Table 2 for the corresponding parameter values). The solid, black circle indicates an alternative, but not chemically feasible minimum for the angle *γ*. The blue, orange, and yellow curves show *χ*_X_^2^, *χ*_D_^2^, and *χ*_J_^2^, respectively. (b) Two-dimensional plot showing *χ*_tot_^2^ as a function of *r*_12_ and *J*. The global minimum of *χ*_tot_^2^ is marked by a solid, red circle. The red line marks the ellipse fitted to the 1.25*χ*_min_^2^ level. The minima of *χ*_X_^2^, *χ*_D_^2^, and *χ*_J_^2^ are also shown (open, red circles). From one contour line to the next, the value of *χ*_tot_^2^ increases by 25%.

Once the simulated annealing algorithm had found a reasonable match between simulation and experiment, the simulation was further improved by adjusting the eight fitting parameters individually such that *χ*_tot_^2^ was minimal for all of them. Plots of *χ*^2^ as a function of *α*, *β*, *γ*, *η*, *ξ*, *r*_12_, *J*, and *g*_*x*_ are shown in Fig. 4 for AMUPol in glycerol/water. The *χ*^2^-curves for the other bis-nitroxides are shown in Fig. SI-3a–l (ESI†). Analysis of the shape of a minimum in *χ*^2^ provides an estimate for the uncertainty in a parameter. These uncertainties are reported in Table 2. The 2D plot of *χ*_tot_^2^ as a function of *r*_12_ and *J* shows an elliptical shape for the minimum (Fig. 4b), which means that these two parameters can, to some extent, compensate each other. For example, an increase in distance does not result in a reduced quality of the fit, if it is accompanied by a stronger exchange interaction.

The right most column in Table 2 reports the microwave on/off enhancement factors observed in DNP experiments at 500 MHz/330 GHz. In the glycerol/water matrix, the enhancements are systematically higher (by about a factor of 4) than in the DMSO/water matrix. In DMSO/water, BTurea gives the highest enhancement, 55. With BTamide-py the enhancement is lower than with BTamide. In glycerol/water, PyPol and AMUPol perform the same within experimental error with enhancements of 190 and 185, respectively. The enhancement with PyPoldiMe is with 145 clearly lower.

## Discussion

We have used multi-frequency EPR spectroscopy to determine the conformations of six bis-nitroxide polarizing agents in their native DNP environment, *i.e.* in a frozen glassy matrix. We analyzed the lineshapes of EPR spectra at three microwave frequencies (9.7, 140 and 275 GHz) simultaneously by means of an advanced fitting routine and extracted, *via* the electron-electron dipolar interaction, the nitroxide-nitroxide distance, *r*_12_, the relative orientation, *α*, *β*, *γ*, and position, *η*, *ξ*, as well as the Heisenberg exchange interaction, *J*. The simulations match the experimental spectra well, which increases confidence in the structural parameters obtained. A blind test of the fitting routine between JS and GM resulted in simulations of high quality and reproduced the structural parameters of a hypothetical bis-nitroxide well within the uncertainty range estimated from the *χ*^2^ curves, see Fig. SI-4 (ESI†).

The method of analysis used here was developed by Hustedt *et al.*^49^ and in 2008 applied by Hu *et al.*^26^ to investigate the conformations of bis-nitroxide polarizing agents available at the time, including fully ^2^H- and ^15^N-labeled BTurea. Remarkably, the structural parameters reported by Hu *et al.* for BTurea do not fully align with ours, particularly the angles *α* and *β* deviate. A possible explanation for this deviation could be the use of an approximate spin Hamiltonian by Hu *et al*., which may not provide an accurate description of the spectra at high magnetic field. Gafurov *et al.* have determined the relative orientation and distance of the nitroxide moieties for the rigid bis-nitroxide polarizing agent bTbK, which has a negligible exchange interaction, by simulation of its EPR spectra at X- and G-band (180 GHz).^60^ Recently Gast *et al.* have attempted a global analysis of the EPR spectra of AMUPol at X, W (94 GHz), and J band, but without the use of simulated annealing the quality of the simulations left room for improvement.^61^

The multi-frequency approach has been instrumental in obtaining the high-quality simulations in this work. For the mono-nitroxides, the D-band spectra revealed small variations in the values of *χ*_*x*_ (Fig. 2b), while the X-band spectra are most sensitive to variations in *A*_*z*_. In the analysis of the bis-nitroxide spectra, the shape of the *χ*^2^-curves depends on the microwave frequency (Fig. 4 and Fig. SI-3, ESI†). The minima in the angles are generally better defined at D and J band. This is most extreme for *γ*, the *χ*_X_^2^-curve at X band is almost flat for BTamide-py, BTurea, and PyPol. On the other hand, the X-band spectra are essential to fix the parameter *r*_12_. For *r*_12_ and *J*, the minima at the three microwave frequencies do not line up and 2D-plots of *χ*_tot_^2^ as a function of *r*_12_ and *J* were necessary to determine the global minimum. The ellipsoidal shape of the minima in these plots shows that the effects of *r*_12_ and *J* on the spectra can partially compensate each other.

**Fig. 5:**
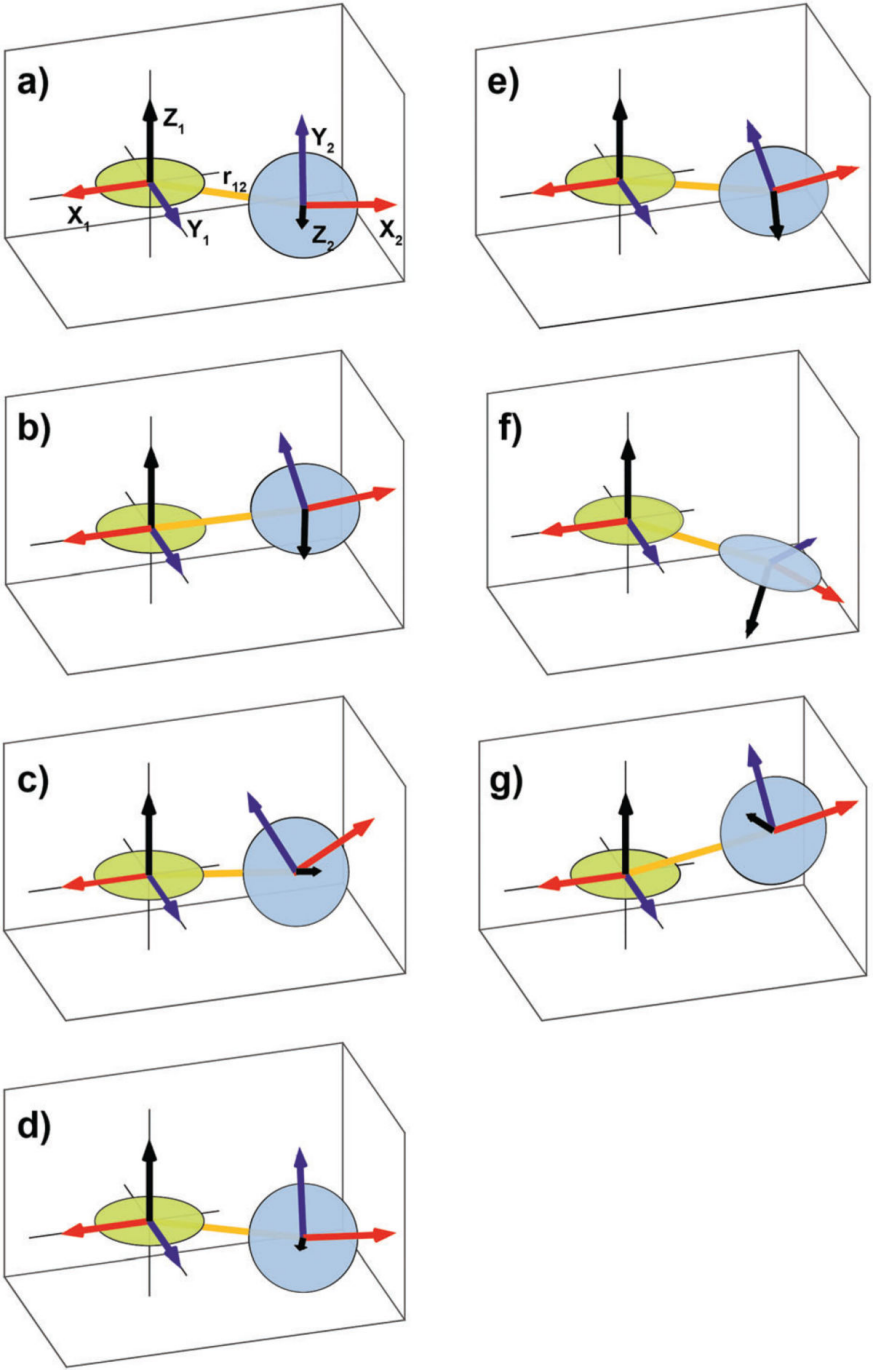
Graphical representation of the relative orientations, *α*, *β*, *γ,* the relative positions, *η*, *ξ*, and the interelectron vector, *r*_12_, of the two nitroxide moieties of (a) BTamide, (b) BTamide-py, and (c) BTurea in DMSO/water, (d) BTurea, (e) PyPol, (f) PyPoldiMe, and (g) AMUPol in glycerol/water. The disks approximate the CN(O)C planes.

The conformations of the six bis-nitroxides are illustrated in Fig. 5. The angles *α*, *β*, *γ*, *η*, and *ξ* are remarkably similar for BTamide, BTamide-py, BTurea, and PyPol (see also Table 2). In spite of the different chemical linkers (amide vs. urea), the conformations of BTamide (a) and BTurea in glycerol/water (d) as well as BTamide-py (b) and PyPol (e) bear a particularly strong resemblance. This suggests that the tetrahydropyran rings codetermine the relative orientation and position of the TEMPO rings. Moreover, the EPR spectra of BTamide-py and PyPol closely resemble each other (Fig. 3), which is reflected in their similar electron-electron distances (11.1 and 11.0 Å) and comparable, yet fairly small *J*-values (−10.1 and −11.2 MHz). The tetrahydropyran rings in BTamide-py and Pypol possibly cause an unfavorable relative orientation of the two TEMPO rings with little overlap of the electronic wave functions. The EPR spectra of BTamide and BTurea in glycerol/water bear less resemblance, because BTamide has a deviant, short electron-electron distance (10.4 Å). Perhaps a rotation around the amide bond brings the two nitroxides relatively close in this bis-nitroxide. The conformation of BTurea in DMSO/water (c) differs slightly from the conformation in glycerol/water (d). This emphasizes that the solvent matrix affects the conformation. In terms of the angles, PyPoldiMe (f) (relatively large *β*, but small *α* and *γ*) and AMUPol (g) (relatively small *β*, small *α* and small *ξ*) stand out. It seems likely that the substituents on the linkers of PyPoldiMe and AMUPol cause these unusual orientations.

Sauvée *et al.* have investigated the conformations of the urea-linked bis-nitroxides BTurea, PyPol, PyPoldiMe, and AMUPol with DFT calculations.^17,19^ All four favor the *trans-trans* conformer (as depicted in Scheme 1) and the angles *θ* between the mean CN(O)C planes of the nitroxide moieties are reported to be 51°, 56°, 90°, and 64°, respectively. Given that the principal *z*-axis of the *g*- and *A*-tensors is approximately perpendicular to this plane, the angle *θ* corresponds to the Euler angle *β* reported here (Table 2). The quantitative agreement between the angle *θ* (from DFT calculations) and the angle *θ* (from multi-frequency EPR) is good for AMUPol (*β* = 54°) and fair for BTurea (*β* = 64° in DMSO/water and *β* = 71° in glycerol/water). For BTurea, the better quantitative agreement with the EPR data in DMSO/water might be arbitrary, since the DMSO/water or glycerol/water matrix was not explicitly taken into account in the DFT calculations (the PCM model was used to include water). While for PyPol and PyPoldiMe the quantitative agreement is not good, both methods result in a similar *β*(*θ*) trend across the four urea-linked bis-nitroxides. In particular for PyPoldiMe, both methods agree that the angle *β*(*θ*) is clearly larger than for the other three bis-nitroxides (*β* = 125°). Sauvée *et al.* suggest that the large angle *β*(*θ*) is the result of the urea-linker becoming nonplanar to accommodate the two methyl groups bound to the N-atoms. For PyPol, the reason for the disagreement *(β* = 97°) could be the poorly defined minimum in *χ*^2^ for *β* (see Fig. SI-3i, ESI†), which suggests a large uncertainty in its value.

From the same DFT calculations, Sauvèe *et al.* report the electron-electron distances, defined as 1/*R*_ee_^3^ = (1/*R*_OO_^3^ + 1/*R*_nn_^3^)/2, for BTurea, PyPol, PyPoldiMe, and AMUPol: 11.6, 11.5, 11.2, and 11.6 Å, respectively. These distances are similar to the distances found here, see Table 2. For PyPoldiMe, this distance matches with the EPR data to 0.1 Å. For the other three bis-nitroxides, the distance from DFT is 0.5 Å larger. To this point we note that the use of the point-dipole approximation in the EPR spectra analysis can introduce a small error in the calculated distance.^47^ Nevertheless, we draw the tentative conclusion that in the frozen DNP matrix BTurea, PyPol, PyPoldiMe, and AMUPol take on the *trans-trans* conformer, as the electron-electron distances would be significantly shortened in the *cis-trans* conformer.^19^

Several authors have used solution (room-temperature) EPR spectroscopy at X band to obtain information on the exchange interaction of bis-nitroxide polarizing agents.^19,28,62^ Sauvée *et al.* found values for *J* of ±32.5, ±22.0, ±22.4, and ±22.1 MHz for BTurea, PyPol, PyPoldiMe, and AMUPol, respectively, in water at room temperature (note that Sauvée *et al.* report J=2J in Gauss). These values are larger, by up to a factor of 2, than the values we find for these bis-nitroxides in a frozen solvent matrix (Table 2). This observation underlines that solution EPR can merely provide an estimate for the exchange interaction in a bis-nitroxide polarizing agent under cryogenic DNP conditions. The analysis of the solution EPR spectra of bis-nitroxides requires the use of a dedicated relaxation superoperator to reproduce the effects of a moving, flexible linker.^19,63,64^ This motion is largely restricted in a frozen solvent matrix. Moreover, conformation(s) observed at room temperature in solution might not be relevant at DNP temperatures. Upon sample freezing, time scales for motion change and the conformations in the eventual frozen glassy matrix are determined by the conformational space at the glass-transition temperature.

In the spectra of the mono-nitroxides (Fig. 2), line broadening reflects variations in the molecular structure of TEMPONE(-py). Besides an isotropic line broadening factor of 0.8 mT, inclusion of *g*-strain improved the quality of the simulations of the mono-nitroxides at D band (Fig. 2b and Table 1). *g*-Strain, or, more precisely, the effect of a distribution of *g*-values, is easily incorporated into the spectra calculations in EasySpin without increasing the computation time. The size of the *g*-strain factor, *f,* showed a small dependence on the ring substituents and on the solvent matrix. For proxyl radicals, the values of *g_x_* and *A_z_* depend on whether the nitroxide carries zero, one, or two hydrogen bonds.^54–57^ Within one frozen solution sample, all three situations can occur simultaneously. The fractions of the three populations depend on the local proticity, which is determined by the chemical structure of the nitroxide radical and the solvent matrix.^58^ It seems plausible that for TEMPONE(-py) a similar effect occurs, giving rise to the observed *g*-strain. In addition, in single crystals, TEMPONE is known to experience interconversion between two twisted crossover conformations of the ring, down to temperatures of 190 K.^63,64^ In an asymmetric environment, *e.g.* a solvent matrix, these two conformations could have different values of *A_z_* and *g*_*x*_, and contribute to *g*-strain.

The simulations of the bis-nitroxide spectra at D and J band were improved by allowing *g_x_* to vary during the fitting procedure. All bis-nitroxides showed an increase in *g_x_* of 0.002–0.003 compared to their mono-nitroxide constituents, except for AMUPol, which showed a larger increase of 0.0045 (Tables 1 and 2). Values of *g_x_* are known to vary slightly between, for example, TEMPO, TEMPOL, 4-amino-TEMPO, and TEMPONE. Hence, we suspect that values of *g_x_* are simply altered by assembling the mono-nitroxides into bis-nitroxides. Why the increase is particularly large for AMUPol, is not clear. The simulations of the bis-nitroxides at D and J band also benefited from the inclusion of *g*-strain (Fig. 3b, c and Table 2). For BTamide, BTamide-py, and BTurea both in DMSO/water and in glycerol/water, the *g*-strain factor is same as for the mono-nitroxide constituents. For PyPol, PyPoldiMe, and AMUPol, the *g*-strain factor had to be increased from 0.07 to 0.09, 0.10, and 0.13, respectively. X-band spectra are not affected by small changes in *g_x_* or inclusion of *g*-strain. However, for BTurea, PyPol, and AMUPol the quality of the simulations at X band improved at the low- and high-field edges when an additional isotropic, Lorentzian line-broadening was included. Possible origins of this line broadening could be strain in *r*_12_, *J*, and *A*_*z*_.

In urea derivatives, the rotation barrier is about 10 kcal mol^−1^ for the C(sp^2^)-N bonds and 2–5 kcal mol^−1^ for the C(sp^3^)-N bonds in urea.^19,65^ Rotational flexibility around the N-C bond connecting the TEMPO moieties to urea would mostly affect *J*, without changing the distance *r*_12_, and the Euler angle *β* and lead to distributions of these parameters in the frozen solvent matrix. Such rotational flexibility offers a possible explanation for the additional *(i.e.* compared to the mono-nitroxides) line broadening we observe for the four urea-linked bis-nitroxides. It is tempting to speculate that any further additional line broadening, which we observe most prominently for AMUPol, is due to other forms of conformational flexibility, possibly enabled by (linker) substituents.

To enable a proper comparison, the DNP experiments at 500 MHz/330 GHz were all done at a concentration of 10 mM and in solvent matrices in which the bis-nitroxides are soluble up to this concentration. At 10 mM, PyPoldiMe in glycerol/water is on the edge of its solubility, which might explain why PyPoldiMe in our hands did not give the superior enhancements reported by others (*ε* = 145).^19,20^ Curiously, also the difference in enhancement between PyPol (*ε* = 190) and AMUPol (*ε* = 185) is not significant in our experiments.^17^ The enhancements with PyPol, PyPoldiMe, and AMUPol, which were tested in glycerol/water, are however much higher than with BTamide (*ε* = 33), BTamide-py (*ε* = 22), and BTurea (*ε* = 55), which were tested in DMSO/water. The difference between PyPol and BTamide-py is particularly striking, since their conformations (from EPR) are virtually the same. Most probably the solvent methyl groups of DMSO adversely affect the observed bulk ^1^H DNP enhancement *via* an increase of the nuclear relaxation rates and the rate of dephasing of the electron-spins.^66,67^ The enhancements from BTamide and BTamide-py are also modest compared to BTurea, even if a correction of 20–30% is applied to account for a fraction of mono-nitroxides in the sample (Fig. SI-2, ESI†). BTamide has a strong *J* and a short *r*_12_, while in BTamide-py the methyl groups have been replaced by tetrahydropyran rings - properties that are expected to aid the DNP efficiency.^14,16,20^ Perhaps the effect of methyl-group replacement becomes negligible if the solvent matrix itself contains methyl groups, but this offers no explanation for the overall disappointing performance of the amide-linked bis-nitroxides.

What is the optimal relative orientation (*α*, *β*, *γ*) of two nitroxides in a cross-effect DNP polarizing agent? Is there a single preferred orientation or a range of orientations for which CE DNP is efficient? In static (non-MAS) CE DNP, the two TEMPO moieties in a bis-nitroxide must be oriented such that when microwave irradiation is applied resonant with nitroxide **1**, the effective EPR resonance frequency of nitroxide **2** differs from that of nitroxide **1** by the ^1^H Larmor frequency. In MAS, this condition is somewhat relaxed, since the matching of the microwave frequency with the energy-level splitting of electron **1** and the CE matching may now occur consecutively (during microwave and CE level crossings) instead of simultaneously.^9,10,32^ As a consequence, the electron-electron interaction, the electron-^1^H dipolar interaction, and electronic relaxation rates will start to play a role and numerical simulations are the only way to determine which relative orientations lead to the highest enhancements in CE DNP/MAS NMR. Perras *et al.* recently used such simulations to investigate this matter, relying on estimated structural parameters *η*, *ξ*, *r*_12_, *J*, *g*_*x*_, and relaxation rates.^29^ The authors conclude that the *y*-principal-axes of the two nitroxide *g*-tensors must be approximately orthogonal, but unfortunately do not assign the principal axes of the *g*-tensor to the molecular structure of TEMPO correctly. Hence, at the moment, a discussion of the effect of the relative orientations on the DNP performance of the six bis-nitroxides is not possible. We hope that by combining existing simulation tools with the structural parameters determined in this work, this question can be addressed some time soon. It would be highly interesting to find out whether the unusual orientations of the nitroxide moieties in AMUPol and PyPoldiMe are responsible for their reported superior DNP enhancements.

## Conclusions

Multi-frequency EPR spectroscopy enabled us to determine the conformations of six bis-nitroxide polarizing agents in the frozen glassy matrix used in DNP/MAS NMR experiments. The EPR spectra displayed complex, yet clearly distinct patterns and were analyzed with an advanced fitting routine to extract the electron-electron dipolar interaction, the Heisenberg exchange interaction, and the relative orientation and position of the two nitroxide moieties. These structural parameters play a critical role in the performance of a bis-nitroxide as a polarizing agent for CE DNP and, hence, the results and the method reported here are important for polarizing agent design. Further optimization of the chemical structure of polarizing agents and the DNP matrix will continue to increase the efficiency of CE DNP and thereby the sensitivity of MAS NMR.

## Supplementary Material

Supplementary Material

## Figures and Tables

**Table 1:** Spin-Hamiltonian parameters used to simulate the frozen solution EPR spectra of mono-nitroxides TEMPONE and TEMPONE-py at X and D band. An isotropic, Gaussian line broadening of 0.8 mT was included as well as a *g*-strain factor, *f*. The values of *f* were 0.09 for TEMPONE in glycerol/water, 0.08 for TEMPONE in DMSO/water, 0.07 for TEMPONE-py in glycerol/water, and 0.08 for TEMPONE-py in DMSO/water. The principle values of the hyperfine tensors are given in MHz

	TEMPONE		TEMPONE-py	
	Glycerol/water	DMSO/water	Glycerol/water	DMSO/water
*g_x_*	2.00847	2.00868	2.00883	2.00893
*g_y_*	2.00605	2.00615	2.00615	2.00615
*g_z_*	2.00215	2.00215	2.00215	2.00215
*A_x_*	12.5	12.5	12.5	12.5
*A_y_*	12.5	12.5	12.5	12.5
*A_z_*	104	101	99	97

**Table 2:** Results from multi-frequency EPR analysis of the frozen solution spectra of six bis-nitroxide polarizing agents^a^

	*α*	*β*	*γ*	*η*	*ξ*	*r*_12_ [Å]	*J* [MHz]	*g_x_*	*f*	Linewidth [mT] [FWHM_Gaussian_ FWHM_Lorentzian_]	DNP *ε*_on/off_
BTamide	**72** ± 14	**77** ± 41^b^	**91** ± 56	**164** ± 16	**100** ± 19	**10.4** ± 0.3	**−17.5** ± 4	**2.00890** ± 0.0006	0.08	[0.8 0.0]	**33** ± 2
BTamide-py	**72** ± 15	**97** ± 95^b^	**105** ± 24	**165** ± 21	**83** ± 39	**11.1** ± 0.5	**−10.1** ± 5	**2.00928** ± 0.0005	0.08	[0.8 0.0]	**22** ± 1
BTurea (DMSO/water)	**89** ± 20	**64** ± 23	**116** ± 14	**180** ± 38	**97** ± 27	**11.0** ± 0.4	**−18.8** ± 4	**2.00900** ± 0.0004	0.08	[0.8 0.3]	**55** ± 5
BTurea (glycerol/water)	**71** ± 31	**71** ± 50^b^	**93** ± 31	**165** ± 20	**98** ± 28	**11.1** ± 0.4	**−17.4** ± 4	**2.00870** ± 0.0005	0.09	[1.0 0.3]	—
PyPol	**76** ± 27	**97** ± 40^b^	**107** ± 24	**160** ± 85	**93** ± 57	**11.0** ± 0.5	**−11.2** ± 4	**2.00910** ± 0.0002	0.09	[0.8 0.3]	**190** ± 10
PyPoldiMe	**54** ± 20	**125** ± 18	**48** ± 35	**164** ± 39	**109** ± 15	**11.3** ± 0.5	**−19.2** ± 5	**2.00910** ± 0.0004	0.10	[0.8 0.0]	**145** ± 5
AMUPol	**52** ± 15	**54** ± 19	**118** ± 55	**161** ± 25	**71** ± 30	**11.1** ± 0.4	**−15.3** ± 4	**2.00928** ± 0.0005	0.13	[0.8 0.3]	**185** ± 5

a Angles are given in degrees. In the point-dipole approximation, the dipolar coupling constant is given by *D* = 5.204 × 10^−20^ Hz/*r*_12_^3^, *i.e.* for AMUPol the dipolar coupling constant is 38.1 MHz. BTamide, BTamide-py, and BTurea were investigated in DMSO/water, BTurea, PyPol, PyPoldiMe, and AMUpol were investigated in glycerol/water.

b For these parameters, the error *χ*_tot_^2^ did not reach the level 2.25*χ*_min_^2^ and uncertainties were estimated by eye from the width of the shallow minimum (see Fig. SI-3a, c, g and i, ESI).

## Footnotes

† Electronic supplementary information (ESI) available. See DOI: 10.1039/c8cp05236k
