# Supplementary material for: Conformation of bis-nitroxide polarizing agents by multi-frequency EPR spectroscopy
Source: Phys Chem Chem Phys. Author manuscript; Available in PMC 2020 May 29. (PMC7256712; doi:10.1039/c8cp05236k)
Supplement: Supplementary Material [file NIHMS1589226-supplement-Supplementary_Material.pdf]

Electronic Supplementary Material (ESI) for Physical Chemistry Chemical Physics.
This journal is © the Owner Societies 2018

SUPPORTING INFORMATION

Conformation of Bis-nitroxide Polarizing Agents by Multi-frequency EPR Spectroscopy

Janne Soetbeer,[a†] Peter Gast,[b] Joseph J. Walish,[c] Yanchuan Zhao,[c] Christy George,[a] Chen Yang,[a] Timothy M. Swager,[c] Robert G. Griffin,[a] and Guinevere Mathies[a*§]

[a.] Francis Bitter Magnet Laboratory and Department of Chemistry, Massachusetts Institute of Technology, Cambridge, MA 02139, USA.

[b.] Department of Physics, Huygens-Kamerlingh Onnes Laboratory, Leiden University, PO Box 9504, 2300 RA Leiden, The Netherlands.

[c.] Department of Chemistry, Massachusetts Institute of Technology, Cambridge, MA 02139, USA.

[*] To whom correspondence should be addressed.

† Current address: Laboratory of Physical Chemistry, Department of Chemistry and Applied Biosciences, ETH Zurich, Vladimir-Prelog-Weg 2, 8093 Zürich, Switzerland

[§] Current address: Department of Chemistry, University of Konstanz, 78464 Konstanz, Germany

Contents

Alignment of J- and D-band spectra	S2
Subtracting the mono-nitroxide contributions for BTamide and BTamide-py	S3
Fitting error plots	S4
Simulation of bis-nitroxide X	S6

Alignment of J- and D-band spectra

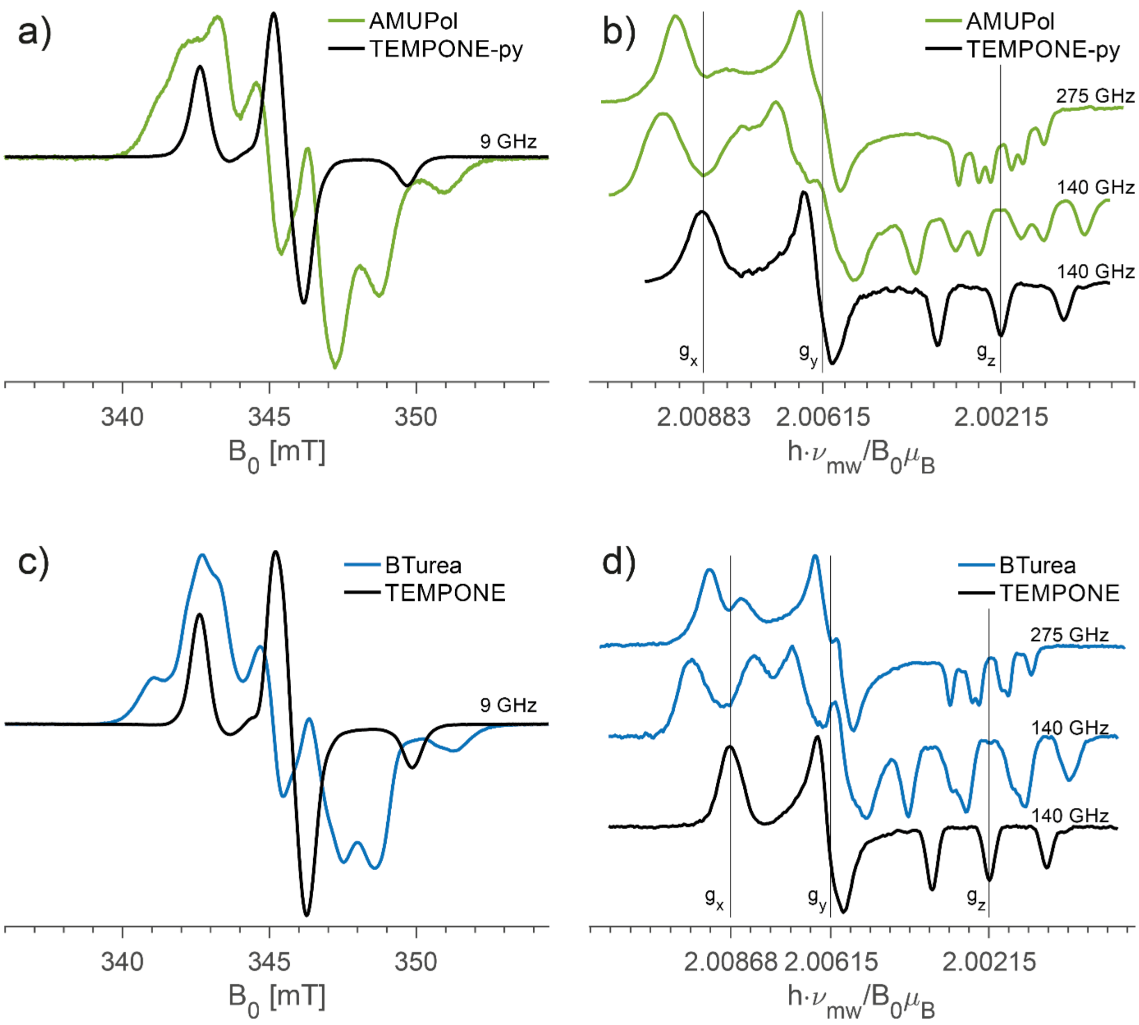

Figure SI-1: EPR spectra of AMUPol in glycerol/water (a,b) and BTurea in DMSO/water (c,d) at X, D, and J band plotted together with spectra of their corresponding mono-nitroxides. On the D-band spectrometer, the field positions are cross-checked during the field sweep with the ^{1}H NMR resonance from a water sample located near the actual sample, which allows accurate determination of the g-values. In (b) and (d), D- and J-band spectra are plotted with the g-values on the x-axis. The J-band spectra are aligned with the D-band spectra at g_z.

Subtracting the mono-nitroxide contributions for BTamide and BTamide-py

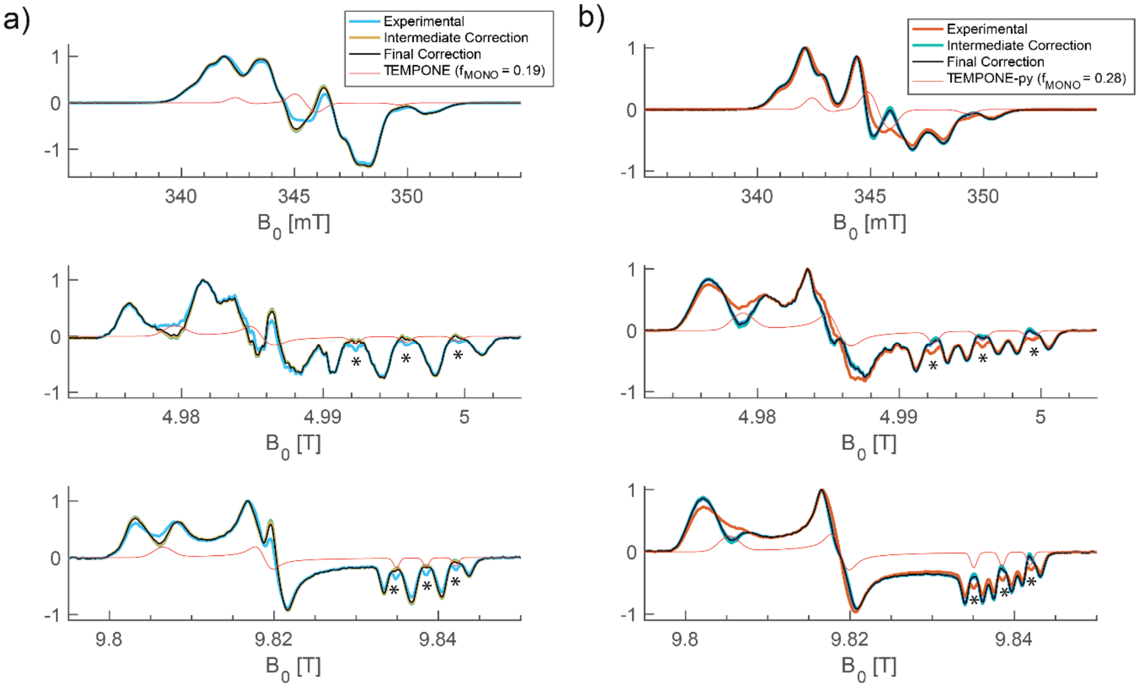

Figure SI-2: Illustration showing subtraction of mono-nitroxide contributions to the X, D, and J-band spectra of BTamide (a) and BTamide-py (b) in DMSO/water. We suspected that the features in the D- and J-band spectra marked with * were due to a fraction of one-sided reduced BTamide(-py) present in the samples. This was confirmed by a J-band spectrum (not shown) recorded on a BT-amide-py sample that had been stored at room temperature for several weeks, which showed a marked increase of all features associated with the mono-nitroxide. Before entering the fitting procedure the mono-nitroxide fractions were quantified and subtracted from the BTamide and BTamide-py spectra in an iterative process. First, the simulated TEMPONE(-py) spectra in DMSO/water (Figure 2 in the main manuscript) were subtracted from the experimental spectra of BTamide(-py) with an estimated fraction f_{MONO}. The resulting spectra entered the fitting routine to obtain simulations of BTamide(-py). The sums of the simulated BTamide(-py) and TEMPONE(-py) spectra were compared to the original experimental spectra to optimize f_{MONO}. The simulated TEMPONE(-py) spectra were then resubtracted from the experimental spectra with the optimized f_{MONO} and the resulting spectra entered the fitting routine again. This procedure was repeated until no changes in f_{MONO} were observed.

Fitting error plots

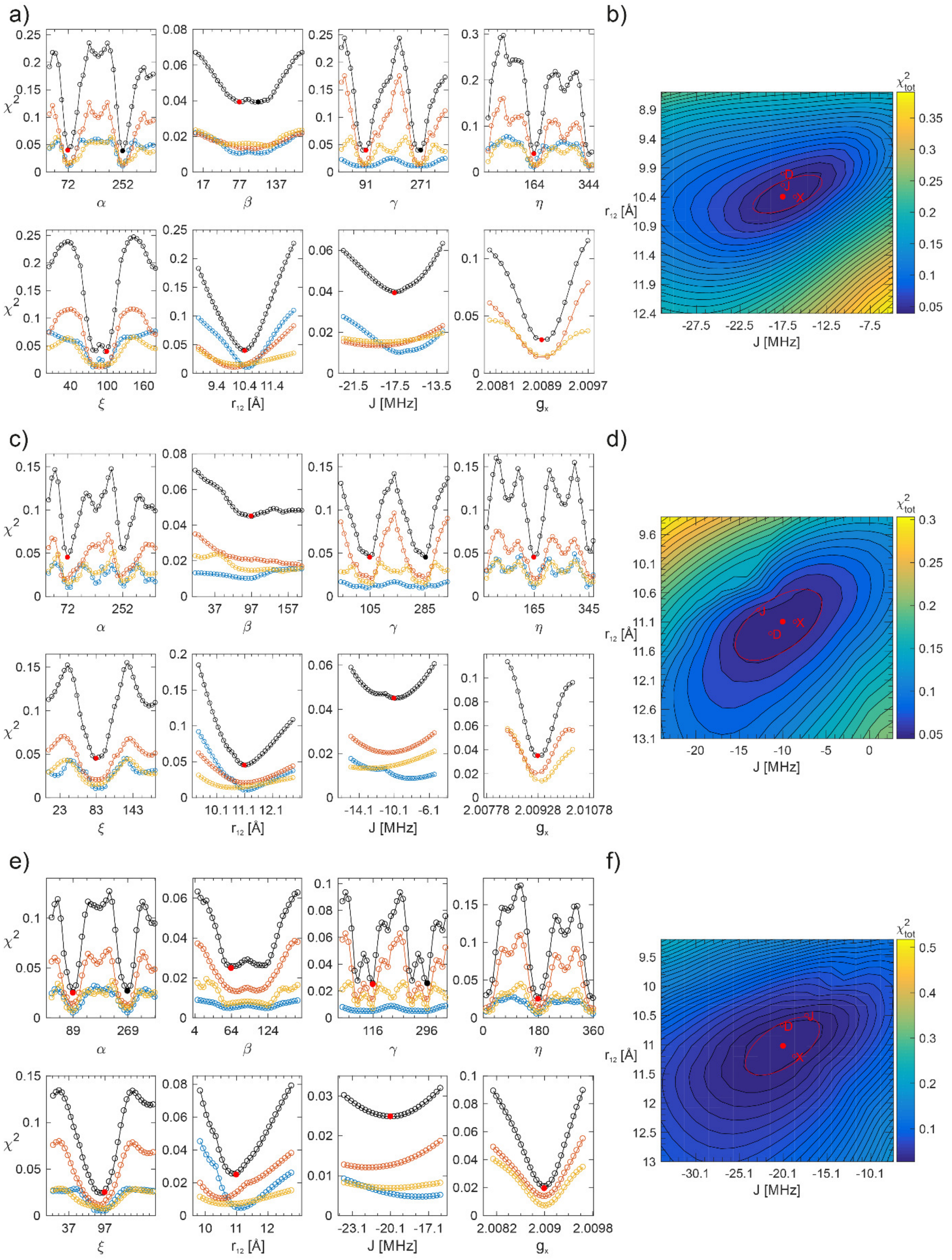

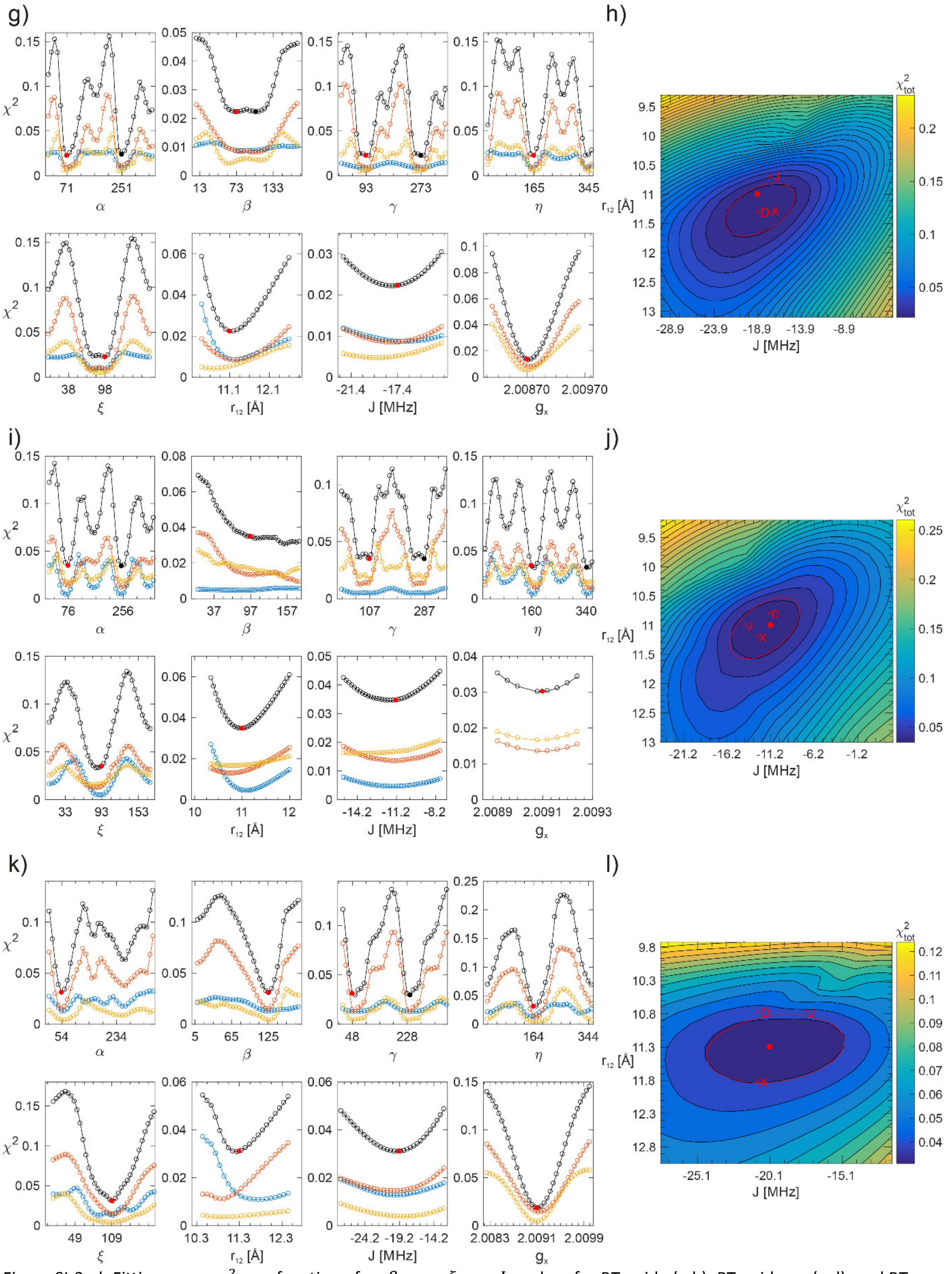

Figure SI-3a-l: Fitting errors χ^2 as a function of α, β, γ, η, ξ, r_{12}, J, and g_x for BTamide (a,b), BTamide-py (c,d), and BTurea (e,f) in DMSO/water and for BTurea (g,h), PyPol (i,j), and PyPoldiMe (k,l) in glycerol/water. The black, blue, orange, and yellow curves show χ^2_{tot}, χ^2_X, χ^2_D, and χ^2_J, respectively. The solid, red circles indicate the global minima of χ^2_{tot} (minimum values of χ^2_{tot} are 0.039, 0.045, 0.025, 0.022, 0.035, 0.031, respectively), the solid, black circles indicate alternative, but not chemically feasible minima. For BTamide and BTurea in DMSO/water and glycerol/water, respectively, where all three angles α, β, γ display double minima, possible combinations of minima were tested systematically to determine which parameter set produced the best fit. In the 2D plots, the minima of χ^2_X, χ^2_D, and χ^2_J are marked by red, open circles.

Simulation of bis-nitroxide X

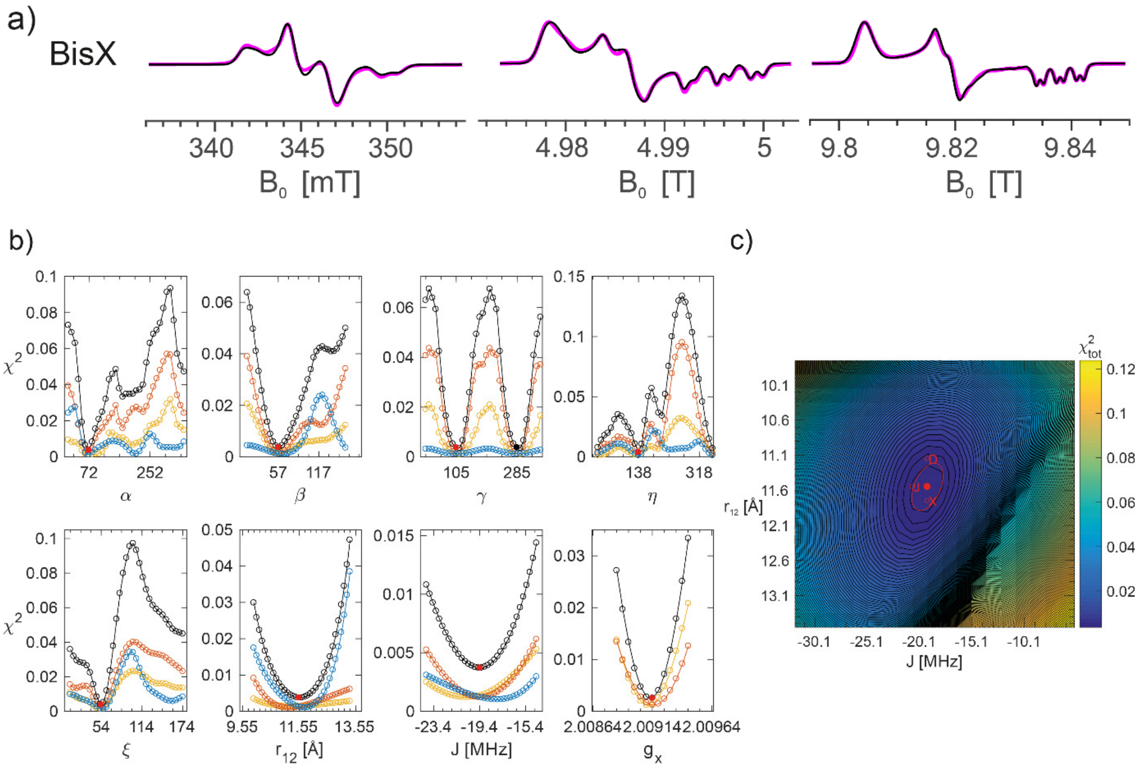

Figure SI-4a-c: (a) Calculated spectra (magenta) at X, D and J band of hypothetical bis-nitroxide X together with simulations (black). (b,c) Fitting errors χ^2 as a function of α, β, γ, η, ξ, r_{12}, J, and g_x for bis-nitroxide X with a minimum value of χ^2_{tot} of 0.0037. The parameters used to calculate the spectra of bis-nitroxide X are listed in Table SI-1 below, together with the starting parameters of the fitting routine and the final parameters used for the simulations in (a).

Table SI-1: Blind-test results of the fitting routine.

	Bis-nitroxide X parameters	Starting parameters fitting routine	Final parameters fitting routine
α	90	65	**72** $\pm$28
β	56	69	**57** $\pm$45
γ	115	91	**105**$\pm$90
η	155	191	**138** $\pm$35
ξ	55	84	**54** $\pm$53
r_{12} [Å]	11.5	10	**11.6** $\pm$0.3
J [MHz]	-18	-8.4	**-19.4** $\pm$1.5
g_x	2.0091	2.0087	**2.0091** $\pm$0.0005
g_y	2.00615	2.0060	2.00615
g_z	2.00215	2.0021	2.00215
A_x	17.5	12.5	17.5
A_y	17.5	12.5	17.5
A_z	98	103.6	99
f	0.08	0.08	0.09
Linewidth Gaussian [mT]	0.6	0.8	0.9
Linewidth Lorentzian [mT]	0.4	0	0
